# Laparoscopic resection of gastric schwannoma: A case report

**DOI:** 10.1016/j.ijscr.2019.10.037

**Published:** 2019-10-25

**Authors:** Fernando Cordera, Andrea Salazar-Vitale, Estephany Mejía-Sánchez, Rodrigo Arrangoiz, David Caba-Molina, Manuel Muñoz-Juárez, Enrique Luque-de-León, Eduardo Moreno-Paquentín

**Affiliations:** a ABC Medical Center, Mexico; b Anahuac University, Mexico

**Keywords:** Gastric schwannoma, Mesenchymal tumor, Laparoscopic

## Abstract

**Introduction:**

Gastric schwannomas are an extremely rare presentation of mesenchymal tumors originating from Schwann cells, accounting for 0.2% of all gastric tumors. Patients are usually asymptomatic, so these tumors are frequently detected incidentally.

**Presentation of case:**

68-year old male patient found to have a 5 cm mass in the lesser curvature of the stomach. After a careful preoperative evaluation, complete laparoscopic resection was performed. Pathology review confirmed a completely resected gastric Schwannoma. The patient’s recovery was uneventful. At a one-year follow-up he remains asymptomatic and with no evidence of disease.

**Discussion:**

We present the uncommon case of a gastric schwannoma that was appropriately treated with a laparoscopic approach and present a current literature review focusing on diagnostic and treatment methods of these rare tumors.

**Conclusion:**

Schwannomas should be included in the differential diagnosis of gastric tumors and can be appropriately treated with a laparoscopic approach.

## Highlights

• This is one of only 220 cases of reported cases of gastric Schwannoma.
• This is one of the very few cases of these rare tumors that has been managed with a laparoscopic approach.
• We present images of the preoperative evaluation (CT) and of the laparoscopic approach (intraoperative photos).
• The patient has over a one-year follow-up and the patient is in perfect condition with no evidence of disease.
• We accompany the case report with a thorough current review on this subject.

## 1 Introduction

Gastric schwannomas are an extremely rare presentation of mesenchymal tumors originating from Schwann cells, accounting for 0.2% of all gastric tumors. Using the Surgical Case Report (SCARE) guidelines [1], we present the uncommon case of a gastric schwannoma that was appropriately treated with a laparoscopic approach and present a current literature review focusing on diagnostic and treatment methods of these rare tumors.

## 2 Case report

**Fig. 1:**
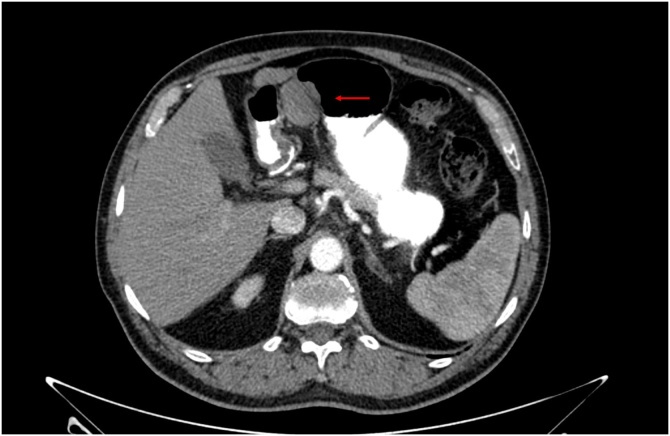
CT with oral and IV contrast. Image shows a mass arising from the lesser curvature of the stomach, which does not enhance with IV contrast.

A 68-year old male with a history of seminoma, currently with no evidence of disease, was referred to our surgical group after a routine check-up abdominal ultrasound (US) revealed a tumor in the lesser curvature of the stomach. An abdominal CT scan with oral and IV contrast confirmed a 5 cm homogenous mass in the lesser curvature of the stomach; there was no suspicious lymphadenopathy and no other suspicious lesions were identified. Patient was completely asymptomatic at the time, referring no weight loss, no changes in diet or appetite, as well as normal GI function. The patient’s case was discussed in a multidisciplinary tumor board and a laparoscopic resection was recommended (Fig. 1).

**Fig. 2:**
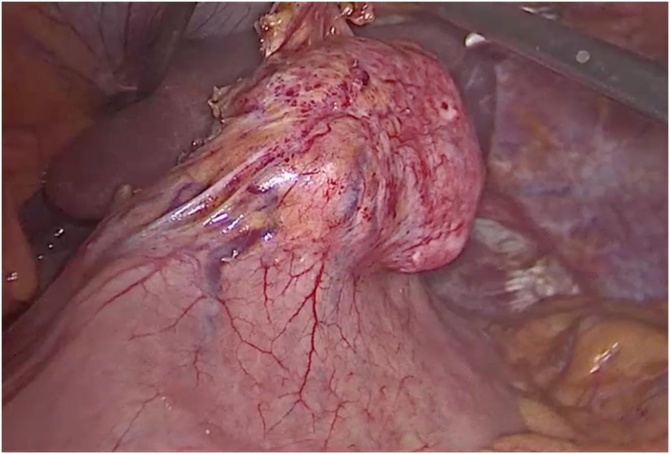
Intraoperative image of the Schwannoma in the lesser curvature of the stomach.

**Fig. 3:**
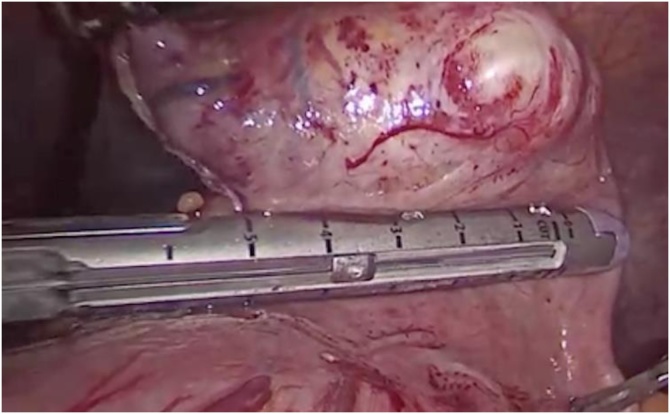
Division of the normal gastric tissue with an endoscopic stapler to resect the Schwannoma with negative margins.

**Fig. 4:**
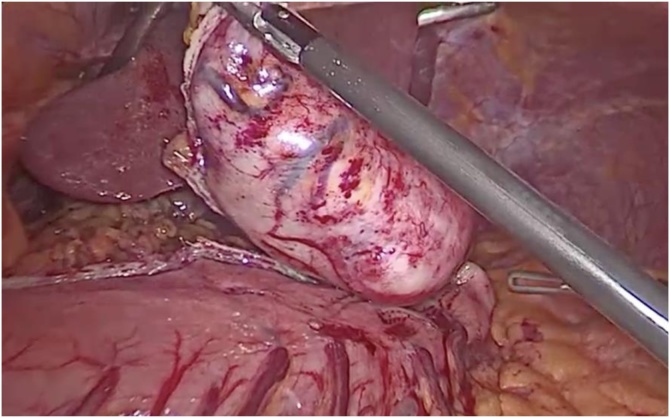
Complete separation of the Schwannoma from the normal stomach.

Under general anesthesia, a laparoscopic exploration confirmed a 5 cm mass in the lesser curvature of the stomach, no suspicious lymphadenopathy and no other lesions were identified. An intraoperative upper GI endoscopy did not identify any other suspicious lesions on the upper GI tract. Using the laparoscopic technique, the gastroesophageal junction was retracted anteriorly and, using a harmonic scalpel, access to the lesser omentum was obtained. Using a 60 mm endoscopic stapler, the tumor was completely resected with 1.5 cm margins, and extracted with an endoscopic bag. Intraoperative pathology confirmed a spindle cell neoplasia with negative margins. A new upper GI endoscopy confirmed that the diameter of the gastric lumen had not been significantly reduced and that the staple line was airtight. No complications were reported. The patient was started on a soft diet the following day. His postoperative recovery was uneventful, and he was discharged home on postoperative day 3 (Fig. 2, Fig. 3, Fig. 4).

The pathology report confirmed a 5.1 × 2.1 cm, lobulated, white-yellow tumor, with well-defined margins. In the immunohistochemical analysis, the tumor was positive for PS100 and SOX10, and negative for CD117 and DOG-1, confirming diagnosis of gastric schwannoma. The tumor was completely resected, and all margins were confirmed clear of tumor. At a one-year follow-up, the patient remains completely asymptomatic and a follow up CT shows no evidence of tumor recurrence.

## 3 Discussion

Schwannomas are rare, benign mesenchymal tumors of the peripheral nerve sheath that arise from the Schwann cells; approximately 221 cases of gastric schwannomas have been reported worldwide [2] and they account for only 0.2% of all gastric tumors [3]. They occur most commonly between the fifth and sixth decades of life and tend to have a higher incidence in the female population [4]. Although most gastric schwannomas are asymptomatic, some patients may experience dyspepsia, early satiety, fullness of stomach, heartburn, or abdominal discomfort as manifestation of the disease [3].

Schwannomas can arise at any point of the GI tract, such as in the stomach, small intestine, colon, or rectum, ranging in sizes from 1 to 12 cm in diameter [5], and represent an uncommon subgroup of submucosal tumors (SMT). In turn, SMTs are subdivided in three categories: neurogenic tumors, such as schwannomas and neurofibromas, myogenic tumors, as is the case with leiomyomas and leiomyosarcomas, and finally, gastrointestinal stromal tumors (GISTs) [6].

Given the fact that most gastric schwannomas are asymptomatic, the diagnosis can represent a challenge for the physician. Most gastric schwannomas are detected incidentally, usually as part of imaging studies performed for other reasons, such as a check-up, as in the patient currently presented. On computed tomography (CT), they tend to appear as homogenous masses, usually showing no signs of necrosis and do not tend to be associated with cystic changes or calcification. Approximately 75% of the masses appear lobulated [7]. Schwannomas can usually be misdiagnosed as GISTs, which can impair treatment selection.

Histologically, gastric schwannomas, like GI leiomyomas and GISTs, are characterized by the presence of spindle cells, with wavy cigar shaped nuclei and collagen structures [8], which can make it difficult to distinguish among SMTs. Moreover, one aspect that can help differentiate among neurogenic tumors of the GI tract is the absence of Antoni A and Antoni B patterns, which are highly common in acoustic neuromas [8].

The definite diagnosis is established by anatomopathological and immunohistochemical analysis after the lesion has been removed [2]. In order to differentiate between gastric schwannomas and other GI tract tumors, an immunohistochemical analysis must be performed. Gastric schwannomas are almost always positive for S100 marker, and very frequently for glial fibrillary acidic protein (GFAP) and CD56. In contrast, GIST are always positive for CD117, CD34 and GIST 1 (DOG-1), which in turn are always negative in gastric schwannomas [9]. Table 1 summarizes the important differences between gastric schwannomas and GIST [10].

**Table 1:** Comparison of characteristics of mesenchymal tumors [9].

Characteristics	GIST	Schwannoma
Average Age	51.6	56.6
Cell of origin	Interstitial cell of Cajal	Schwann cells, enteric plexus
Common localizations	Stomach	Stomach
Other localizations	Small intestine	Colon, esophagus
	Colon, rectum, esophagus	
Macroscopic cut	Grey-pink	White-yellow
Histology	Spindle cell	Spindle cell
	No lymphoid halo	Lymphoid Halo
Immunophenotype		
**S-100**	–	+
Vimentin	+	+
Actinic muscle	Variable	–
Desmin	–	–
GFAP	–	Variable
**CD34**	+	–
Nestin	+	Variable

Once the diagnosis has been established, surgical resection with clear margins remains the treatment of choice [11]. Until now, most schwannomas had been resected using an open approach; however, as this case exemplifies, these tumors can be safely and adequately removed through a laparoscopic approach. As it occurs with other tumors, the laparoscopic approach is associated with the same favorable oncological outcome and is accompanied with the benefits of a shorter hospital stay, reduced postoperative pain, and earlier return to normal activities.

Recently, endoscopic submucosal resection and endoscopic full thickness resection have also been proposed as a non-invasive methods for treating these tumors [8]. However, these procedures remain investigational and currently the laparoscopic approach remains as the treatment of choice [12].

Most gastric schwannomas are located in the body of the stomach, and associated lymphadenopathy is not necessarily indicative of malignancy [7]. Gastric schwannomas do not tend to metastasize to nearby lymph nodes, thus lymphadenectomy is performed only when enlarged lymph nodes are observed [12,13].

A comprehensive review of 137 cases of gastric schwannomas treated surgically yielded no cases of recurrence after a follow-up period ranging from 1 to 336 months [14]. Therefore, it is safe to conclude that benign gastric schwannomas rarely recur, and thus, frequent follow up with CT imaging is not recommended [12]. When recurrence does occur, it is usually associated with incomplete surgical margins as opposed to malignant behavior of the tumor [4]. After an appropriate resection, gastric schwannomas have an excellent prognosis, and a full recovery is observed in the majority of the cases.

## 4 Conclusions

Gastric Schwannomas are benign, slow-growing mesenchymal tumors. Although rare, they should be taken into consideration in the differential diagnosis of any gastric lesion. Definite diagnosis is established through pathological and immunohistochemical analysis. The treatment of choice remains surgical resection with clear margins. The laparoscopic approach is an excellent option for many of these patients. After an appropriate resection the prognosis is excellent, and recurrence is rare.

## Sources of funding

None.

## Ethical approval

Exception: Case report of interesting case encountered during normal medical practice.

## Consent

Written informed consent was obtained from the patient for publication of this case report and accompanying images. A copy of the written consent is available for review by the Editor-in-Chief of this journal on request.

## Author contribution

Fernando Cordera: treating physician, concept and design, data collection, writing.

Andrea Salazar-Vitale: data collection, data analysis, writing.

Estephany Mejía-Sánchez: data collection, writing.

Rodrigo Arrangoiz: data collection, writing.

David Caba-Molina: data analysis.

Manuel Muñoz-Juárez: data analysis.

Enrique Luque-de-León: data analysis.

Eduardo Moreno: data analysis.

## Registration of research studies

N/A.

## Guarantor

Fernando Cordera.

## Provenance and peer review

Not commissioned, externally peer-reviewed.

## Declaration of Competing Interest

None.
